# Research on psychological attributions and intervention strategies of new energy hybrid vehicle purchase behavior

**DOI:** 10.1038/s41598-023-35949-0

**Published:** 2023-06-17

**Authors:** Qing Guo, Wenlan You

**Affiliations:** School of Economics and Trade, Guangdong University of Foreign Studies, Guangzhou, 510006 China

**Keywords:** Environmental sciences, Environmental social sciences

## Abstract

Based on the questionnaire survey, this paper developed a theoretical model of the factors influencing consumers' purchase behavior for new energy hybrid vehicles using the theory of planned behavior and structural equation modeling techniques. It then used SPSS and AMOS to perform factor analysis, model fitness, and path analysis to reach the following conclusions: perceived behavioral control, behavioral attitude, and subjective norm have a significant positive influence on behavioral intention, and behavioral intention has a significant influence on actual behavior. However, there is no significant effect of perceived behavioral control on actual purchase behavior, but rather an indirect effect on actual behavior through the mediating variable of behavioral intention. The analysis of the multi-group model based on the individual characteristics of consumers showed that the coefficient of subjective norm on behavioral intention was higher for extroverted consumers than for introverted consumers; the influence of behavioral attitude on behavioral intention was significantly higher for introverted consumers than for subjective norm on behavioral intention.

## Introduction

China's rapid economic growth in recent years has been accompanied by declining air quality^1^. On the one hand, energy demand and consumption have increased, with China's reliance on foreign oil reaching 72% in 2021. Nonetheless, environmental pollution is a significant issue. 35.7% of China's prefecture-level cities have concerns with ambient air quality exceeding the limit in 2021. One of the main causes of energy consumption and environmental degradation is the sharp rise in car ownership. With an average annual growth rate of 29.38%, China's car population increased from 108 million in 2012 to 395 million in 2021, increasing fuel consumption and exhaust emissions significantly. The problem of air pollution brought by exhaust fumes from cars powered by internal combustion engines (ICEVs) gets worse as China's use of gasoline and diesel rises from 257 million tons in 2012 to 336 million tons in 2021. According to Givord^2^, diesel and gasoline levies lower the demand for conventional vehicles but have no effect on CO_2_ emission intensity. A crucial step in resolving the tension between fuel supply and demand, cutting pollutants, and improving the atmosphere is the development of electric vehicles. New energy cars have been acknowledged as a sustainable solution to minimize gasoline use and emissions in the transportation industry as conventional energy sources become more scarce globally^3^. From the standpoint of a sustainable economic development route and the preservation of the environment on which humanity depends, the development of new energy hybrid cars is of utmost strategic importance. Hybrid vehicles will continue to be the mainstay of energy-efficient vehicles in China for some time to come, despite the fact that new energy hybrid vehicles have many benefits and consumer acceptance of new energy vehicles is growing annually^4^. This is because new energy hybrid vehicles still lack key technologies, such as battery technology and charging infrastructure.

Although enthusiasm for private purchases is still low^5^, new energy vehicle policies have helped China's new energy industry flourish in part^6^. At this point, these laws are being updated and modified to match consumer demand for purchases^7^. In order to increase sales of new energy vehicles, study should be done on this market because the current market share of such vehicles in China is still quite low^8^. The poor figures on the adoption of hybrid vehicles globally may prompt a number of queries, such as: Are hybrid vehicles not appealing enough to customers to win their hearts and minds? When it comes to acquiring these automobiles, do car owners have any particular preferences? In this regard, Alberini et al.^9^ contended that consumer willingness to purchase hybrid vehicles may be triggered by a wide range of motivating factors, including economic considerations, while McLeay et al.^10^ suggested that studying the drivers affecting hybrid vehicle adoption could improve adoption rates.

The promotion of hybrid technology iterations and upgrades is very real and beneficial for reducing carbon emissions in the automotive industry, and it is also a crucial time to promote the use and development of hybrid technology. Hybrid vehicles are less noisy and vibration-free than conventional engines^11^. Now the question is: Why do people buy new energy hybrid cars? Also, what are the psychological underpinnings of consumer behavior and how do these psychological underpinnings influence behavior? How can efficient intervention policies be created to influence consumer purchasing decisions (e.g., how can new energy hybrid car purchasing decisions be maintained)? The theoretical community has not adequately addressed these issues. It takes a mix of various intervention methods to overcome the limits of a single strategic tool when developing appropriate promotion tactics since they must take into account both the internal psychology of customers and the influence of the external environment^12^. Based on the questionnaire survey, this paper developed a theoretical model of the factors influencing consumers' purchase behavior for new energy hybrid vehicles using the theory of planned behavior and structural equation modeling techniques, to clarify the psychological attributions and intervention pathways for the purchase behavior of new energy hybrid vehicles. The goal is to serve as a resource for various organizations to develop green and sustainable development strategies.

## Literature review

A search of the literature reveals that current research by scholars can be broadly classified into three categories.

### Willingness to consume new energy vehicles

Li et al.^13, 14^ took urban residents in Tianjin as an example to study the impact of environmental stimulus and psychological factors on the purchase behavior of new energy vehicles, and concluded that consumers care more about the comfort of the vehicle. Sun et al.^15^ conducted an empirical test on the role of government subsidies on the purchase intention of new energy vehicles using a hybrid logit model and found that price is an important factor in determining whether consumers choose new energy vehicles. Li and Guo^16^ used a binary logit model to study the purchase decision of new energy vehicles among Wuhan residents and found that consumers would focus on safety when purchasing new energy vehicles. Mi et al.^17^ used the exploratory research technique of rooting theory to analyze the factors influencing knowledge-based consumers' new energy vehicle purchase behavior, and found that knowledge-based consumers care more about the supporting service facilities of the vehicle. Huang and Ge^18^ constructed a model of the influence mechanism of electric vehicle purchase intention based on the theory of planned behavior, and concluded that monetary incentives have a significant positive effect on consumers' intention to purchase electric vehicles in Beijing. Hamamoto^19^ empirically investigated the factors influencing consumers' choice to purchase hybrid vehicles and showed that consumers who are more concerned about fuel economy may tend to choose hybrid vehicles.

### Psychological attributions of consumer buying behaviour

Many academics have investigated the psychological attribution of consumer buying behavior from various viewpoints in theoretical and empirical studies. According to empirical study of consumers' green consumption patterns from 2022, the new crown pneumonia pandemic increased consumers' desire to make green product purchases and had a favorable impact on consumer behavior^20^. Price sensitivity, according to Young et al.^21^, inversely attenuated the association between environmental responsibility and green purchasing. According to Young et al.^21^, price sensitivity adversely modifies the association between consumer willingness and environmental responsibility. According to Sheng et al.^22^, the price of green products is higher than the budget for purchasing, which is the main cause of the discrepancy between green consumption behavior and intention. According to Deng and Nam^23^, innovation behavior and consumers' perceptions of risk are positively correlated. Chan and Lau^24^ discovered a significant correlation between eco-emotion, eco-knowledge, and desire to make green purchases as well as actual purchasing behavior. Wang and Wang^25^ investigated the factors influencing Beijing residents' intention to buy new energy vehicles using the Theory of Planned Behavior. They came to the conclusion that purchase attitudes, subjective norms, and perceived behavioral control were the main influences on Beijing residents' intention to buy new energy vehicles.

### Application of the theory of planned behaviour

The Theory of Reasoned Action (TRA) was developed by Icek Ajzen as a successor to the Theory of Reasoned Action (TRA) co-proposed by Ajzen and Fishbein. He expanded TRA to include a new concept of Perceived Behavior Control (PBC) of the self, which developed into a new model of behavioral research, the Theory of Planned Behavior (TPB). TPB is a new model of behavioral research that considers attitudes, subjective norms and perceived behavioral control as the three main variables that determine behavioral intentions. The TPB has been widely used in academia to study a number of behavioral areas. For example, Zeng^26^ studied the factors influencing residents' participation in commercial pension insurance based on the theory of planned behaviour, and the results showed that residents' participation in commercial pension insurance was significantly influenced by the traditional concept of "raising children for old age" and other personal will factors. Guo et al.^27^ constructed a model of driver training based on the TPB and used structural equation modelling (SEM) to capture the complex relationships between variables. Based on the TPB, Li et al.^13, 14^ investigated the mechanism of conversion of virtual tourism experience to field tourism behaviour, and the results showed that virtual tourism experience, perceptual behavioral control significantly influenced virtual tourists' attitudes. Ma et al.^28^ studied the relationship between corporate innovation and founder management based on the extended TPB, and the findings showed that founders' male and highly educated, large firm size and other factors were effective in increasing firms' investment in innovation.

In summary, there is more research on the willingness to consume new energy-powered vehicles and less literature on the willingness to consume new energy-hybrid vehicles, which are clearly different. At the same time, most of the scholars' studies are based on the purchase intention of new energy hybrid vehicles in a particular city and use it to extrapolate the actual situation in China, which is not a representative enough sample. Therefore, this paper refers to scholars' research on the psychological attribution of purchase behaviour, uses the theory of planned behaviour and structural equations to clarify the psychological attribution of new energy hybrid vehicle purchase behaviour and intervention paths based on 358 questionnaires from 10 cities with different degrees of economic development, using the theory of planned behaviour and structural equations to enrich, to a certain extent, the theory of Theoretical research on consumer purchasing behaviour and its intervention strategies.

## Theoretical model and research hypothesis

TPB was developed by Icek Ajzen, according to this theory, behavioral attitude, subjective norm and perceived behavioral control are the three main variables that determine behavioral intention. Therefore, based on the TPB and structural equation model, this paper analyzes the psychological attribution and intervention strategy of new energy hybrid electric vehicle purchasing behavior.

While decision making is considered to be the result of a number of complex factors, the TPB suggests that the most important factor influencing decision making is an individual's behavioral intention, with the separate variable of behavioral intention alone explaining an average of 22% of behavioral change^29^. When considering hybrid car purchases, car users' preferences may determine their attitudes towards the purchase of a car^30^, and attitudes have a significant impact on the willingness to purchase environmentally friendly cars^31^. Regarding consumers' subjective norms and their behavioral intention to purchase new energy-powered vehicles, White and Sintov^32^ found that when people are aware that people in their social circle or around them are decarbonising the transport system by purchasing environmentally friendly vehicles, they tend to be encouraged to 'behave that way'. The higher the social pressure, the more likely people are to be influenced in their behaviour^33^. Based on the theory of planned behaviour and the theory of rational behaviour, Zhang^34^ explored the factors influencing consumers' green product consumption behaviour and showed that there is a mediating effect of perceived behavioral control between environmental concerns and consumer purchase behaviour. In addition, if certain features of the hybrid vehicle fall below their expectations or preferences, it may limit their interest in performing the intended purchase behavior^31^. Therefore, the effect of perceived behavioral control on behavioral intentions to purchase new energy powered vehicles will be further explored in this study.

Therefore, based on the theory of planned behaviour and the findings of the above scholars, this paper proposes the following hypothesis.

- H1: There is a positive relationship between consumers' relevant behavioral attitude and their behavioral intention to purchase new energy-powered vehicles.
- H2: There is a positive relationship between consumers' relevant subjective norm and their behavioral intention to purchase new energy-powered vehicles.
- H3: There is a positive relationship between consumers' relevant perceived behavioral control and their behavioral intention to purchase new energy-powered vehicles.
- H4a: There is a positive correlation between consumers' relevant perceived behavioral control and actual purchase behavior.
- H4b: The relevant perceived control behavior of consumers can influence the actual purchase behavior through the mediating variable behavioral intention.
- H5: There is a positive correlation between consumers' relevant behavioral intention and their actual behavior of purchasing new energy-powered vehicles.

**Figure 1:**
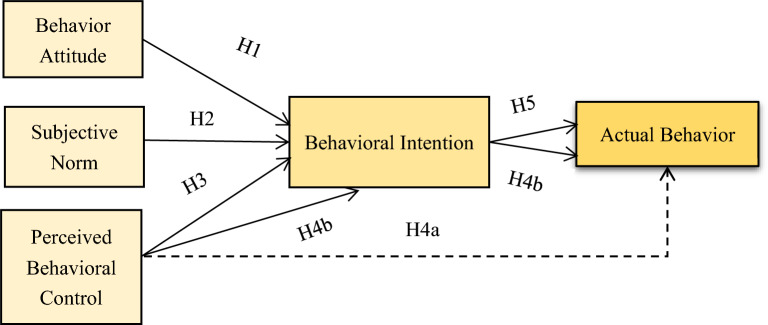
Model of planned behavior theory. Figure depicts the theoretical model of the program in this paper, including behavioral attitudes, subjective norms, perceived behavioral control, behavioral intentions, and actual behavior. Different arrows are used to characterize the research hypotheses H1–H3, H4a, H4b, and H5, respectively.

The precise theoretical model of the scheme is depicted in Fig. 1, where the dotted line represents the hypothesis H5 proposed in this work and two H4b are marked to distinguish them from hypotheses H4a and H5. The two arrows collectively reflect hypothesis H4b (behavioral intention is only used as a mediating variable). According to this conceptualization of the decision-making process, if consumers have a preference or positive attitude toward new energy hybrid vehicles, if they can fully understand and trust the green message delivered by the organization, and if the purchase of new energy hybrid vehicles is a behavior that is socially encouraged and can earn respect from others, then they will have the behavioral intention to buy new energy hybrid vehicles, which may eventually lead to the purchase of a new energy hybrid vehicle.

### Informed consent

Informed consent was obtained from all the subjects involved in the study.

## Data collection and data testing

### Survey methods and results

The five variables behavioral intention, behavioral attitude, subjective norm, perceived behavioral control, and actual behavior are all measured in this study using a survey questionnaire and a five-point Likert scale. The information in this research was gathered by postgraduate students traveling back to their hometowns on National Day in order to gather information because of the diversity of the sources used by university students and the practical necessity for a wide range of respondents in this study. The study is divided into sections according to the concentration of urban commercial resources, urban hubs, lifestyle diversity, and urban people's activity, and the surveyed cities cover Tier 1 to 5 cities, which are widely representative in terms of economic development and population distribution. This ensures that the sample is representative of the population as a whole. Table 1 shows the hierarchy of Chinese cities. Guangzhou and Shenzhen are China's first-tier cities; Zhongshan and Shijiazhuang are China's second-tier cities; Liuzhou and Zhaoqing are China's third-tier cities; Chenzhou and Changde are China's fourth-tier cities; and Yangquan and Hegang are China's fifth-tier cities. The sample was drawn equally, i.e., 40 questionnaires were distributed to each surveyed city, where the number of different types of people with the same physical characteristics was also drawn equally, for example, the ratio of male to female respondents in the same city was controlled as much as possible to 1:1. Specifically, we first classified the target regional population according to factors such as gender, age, income and education. Then, in each classification, a corresponding number of people were randomly invited to focus on filling out the physical questionnaire. A total of 400 questionnaires were distributed and 379 questionnaires were actually collected, with a recovery rate of 94.75%. After excluding some questionnaires with a large amount of missing data or apparently random responses, 358 questionnaires were valid, with an effective rate of 89.50%. The results of descriptive statistics of demographic variables are shown in Table 2.

**Table 1:** China's city hierarchy.

City level	Economic indicators	Representative cities
Tier 1	High	Beijing, Shanghai, Guangzhou, Shenzhen, Tianjin
Tier 2	Upper middle	Chongqing, Chengdu, Hangzhou, Zhongshan, Shijiazhuang
Tier 3	Medium	Changsha, Xiamen, Liuzhou, Zhaoqing, Fuzhou
Tier 4	Lower middle	Haikou, Yinchuan, Urumqi, Chenzhou, Changde
Tier 5	Low	Qiannan, Lijiang, Yangquan, Hegang, Shizuishan

**Table 2:** Descriptive statistics for demographic variables.

Variables	Variable definitions	Effective percentage	Variables	Variable definitions	Effective percentage
Gender	Male	56.14	Income	2999 and below	39.94
	Female	43.86		3000 and above	60.06
Age	29 years and under	37.70	Transport consumption	400 and below	50.27
	30–59 years old	25.43		401 and above	49.73
	60 years and above	36.87	Academic qualifications	Tertiary and below	58.10
				Bachelor's degree and above	41.90

Data source: Authors' statistics based on valid questionnaires.

As can be seen from the data on the demographic variables in Table 2, the sample in this study has a wide coverage and is representative enough for further analysis.

We confirmed that all methods were carried out in accordance with relevant guidelines and regulations, all experimental protocols were approved by the academic committee of Guangdong University of Foreign Studies, and consent was obtained from all subjects and/or their legal guardians.

### Questionnaire design and test results

In addition to the basic information of gender, age and income level, this questionnaire mainly asks consumers about their behavioral attitude, subjective norm, perceived behavioral control, behavioral intention and actual purchase behavior. Table 3 shows the final questionnaire items and the corresponding questions. Questions X36 and X40 were set for behavior intention. Cronbach's α of this dimension was 0.678. X23 and X29 were set for behavior and attitude. Cronbach's α of this dimension was 0.698. X30 and X44 were set for perceived control. Cronbach's α of this dimension was 0.648. X12 and X22 were set for the subjective specification. Cronbach's α of this dimension was 0.722, and actual behavior was set for X27 with one question (Table 3). The Cronbach's coefficients for each latent variable in Table 4 reflect that the scale in this paper has good reliability. For the test of validity, as this paper has previously selected a number of suitable questions for further analysis based on the concept of latent variables included in the theory of planned behaviour, each of which has a corresponding latent variable. Therefore, this paper proceeds directly to the validation factor analysis. For the aggregated validity of the latent variables, this paper refers to scholars Raukov and Wu^35^ and uses AMOS software to conduct a validating factor analysis on 379 valid questionnaires. The results showed that the standardised estimates (Std-Estimate) of the potential variables corresponding to behavioral attitudes, subjective norms, perceptual behavioral control and behavioral intention respectively exceeded the benchmark value of 0.5, and most of the estimates were significant at the level of P < 0.01 (***). The validated factor analysis results showed that the proposed study variables had strong aggregate validity. Based on the results of the validation factor analysis, this paper further calculated AVE (average variance extracted) and CR (combined reliability) according to the formula, and the results are shown in Table 5: the CR values of all latent variables were higher than 0.6, the AVE of behavioral intention and perceptual behavioral control were slightly lower than 0.5, but the AVE of the remaining variables were higher than 0.5. According to Fornell and Larcker, if the AVE is less than 0.5, but the composite reliability is higher than 0.6, then the convergent validity of the construct is still adequate^36^. This indicates that the degree of extraction of the measures within the factors is good. Table 6 demonstrates that the latent variables' square roots of the average variance extracted (AVE) are all bigger than the values of the other latent variables' Pearson correlation coefficients, indicating that they have more outstanding discriminant validity. The survey data in this study can pass the validity test when the results of the aggregated validity and discriminant validity tests are combined.

**Table 3:** Question (variable) setting of the questionnaire.

Variables	Question	Variables	Question
X12	I think the public has a responsibility to promote conscious shopping for new energy hybrid vehicles	X23	I think it is necessary to implement new energy hybrid cars
X22	I think buying new energy hybrid cars is the expectation and requirement of society for people to take environmental responsibility	X29	I think the application of new energy hybrid cars have good prospects
X36	If the government subsidies, I am willing to buy new energy hybrid cars	X44	I am well aware of the subsidy policy related to new energy hybrid vehicles
X40	If the budget is enough, I am willing to buy a new energy hybrid car	X30	In my daily life, I often see new energy hybrid cars
X27	I am willing to use new energy hybrid cars

**Table 4:** Cronbach’ s alpha for each dimension.

Potential variables	Behavioral intentions	Behavioral attitudes	Perceptual behavioral control	Subjective norms
Cronbach’ s alpha	0.678	0.698	0.648	0.722

Data source: Compiled by the authors from the results of SPSS 25.0 software runs.

**Table 5:** Distinct validity tests.

Variables	Average variance extracted AVE values	Combined confidence CR values
Behavioral attitudes	0.534	0.731
Subjective norms	0.567	0.723
Behavioral intentions	0.478	0.698
Perceptual behavioral control	0.479	0.712

Data source: Authors' calculations based on formulae.

**Table 6:** Pearson correlation coefficients and AVE root values.

Variables	Behavioral attitudes	Subjective norms	Behavioral intentions	Perceptual behavioral control
Behavioral attitudes	0.731			
Subjective norms	0.512	0.753		
Behavioral intentions	0.556	0.621	0.691	
Perceptual behavioral control	0.514	0.643	0.527	0.692

The diagonal values are the root values of this factor AVE and the remaining values are Pearson correlation coefficients.

Data source: Authors' analysis based on SPSS 25.0 software.

Common method bias refers to artificial covariation between predictor and effector variables caused by the same data source or rater, the measurement environment, the item context and the characteristics of the item itself. There are two common tests for common method bias: (1) using validated factor analysis, all items analysed are placed in one analysis frame and if the model does not meet the fit indicators such as chi-squared freedom ratio, GFI, RMSEA, RMR, CFI, NFI, NNFI, etc., then the model is a poor fit, i.e. it means that all the measures should not belong to the same factor and therefore indicates that the data pass the common method deviation CMV test and that the data have no common method deviation issues. (2) Exploratory factor analysis EFA: factor analysis was conducted by placing all indicators under one factor, and if the variance explained by the factor was less than 40%, the study data were considered not to have serious common method bias, otherwise, common method bias was considered to exist.

The results of the common method deviation test using method (1) in this study are shown in Table 7. It is easy to see that the indicators did not meet the standard values, so the survey data used in this study can pass the common method deviation test.

**Table 7:** Results of the common method deviation test.

Commonly used indicators	X^2^	df	P	Cardinality ratio of freedom	GFI	RMSEA	RMR	CFI	NFI	NNFI
Judgement criteria	–	–	> 0.05	< 3	> 0.9	< 0.10	< 0.05	> 0.9	> 0.9	> 0.9
Value	318.596	27	0	11.8	0.593	0.169	0.071	0.61	0.593	0.480

Data source: Authors' analysis based on SPSS 25.0 software.

## Analysis of test results

This paper uses structural equation model to test the research hypotheses. RMSEA is a more important indicator in the model fit evaluation system, as can be seen from Table 8, the RMSEA value is 0.027, which meets the fit criteria. Among the absolute fit index, value-added fit index and parsimonious fit index statistics, all of them meet the acceptable criteria of the model except PGFI. At the degree of freedom of 20, the chi-square value of model fitness is equal to 25.290, and the significance probability value is 0.191 > 0.05, indicating that the theoretical model of this paper can fit well with the survey data, and the convergence validity of the model is quite good, which can be used to test the research hypothesis.

**Table 8:** Key indicators for model fitness tests.

Statistical test volume	Criteria for adaptation or critical value	Data test results	Model adaptation judgement
Absolute suitability index			
χ^2^	p > 0.05	25.290 (p = 0.191)	Adaptation
Cardinality ratio of freedom	< 2.00	1.264	Adaptation
RMR value	< 0.05	0.021	Adaptation
RESEA value	< 0.08	0.027	Adaptation
GFI value	> 0.90	0.984	Adaptation
AGFI value	> 0.90	0.965	Adaptation
Value added suitability index			
NFI value	> 0.90	0.969	Adaptation
RFI value	> 0.90	0.944	Adaptation
IFI values	> 0.90	0.993	Adaptation
TLI value	> 0.90	0.988	Adaptation
CFI values	> 0.90	0.993	Adaptation
Minimalist fit index			
PGFI values	> 0.50	0.437	Not applicable
PNFI values	> 0.50	0.538	Adaptation
PCFI values	> 0.50	0.552	Adaptation
CN value	> 200	531	Adaptation

Data source: Compiled by the author from AMOS software runs.

The results of the tests of the model hypotheses are shown in Table 9. The standardized path coefficients of behavioral attitude, perceived behavioral control, and subjective norm on behavioral intentions all reached significant levels, so hypotheses H1, H2, and H3 were valid. Meanwhile, the standardized path coefficients of behavioral intention on actual behavior also reached a significant level, hypothesis H5 was valid. But the standardized path coefficients of perceived behavioral control on actual behavior did not pass the test of data, so hypothesis H4a was not valid.

**Table 9:** Results of model hypothesis testing.

Assumptions	Estimate	Std estimate	P	Conclusion
H1: behavioral attitudes → behavioral intentions	0.492	0.447	***	Support
H2: Subjective norms → behavioral intentions	0.405	0.317	***	Support
H3: Perceptual behavioral control → behavioral intention	0.376	0.398	***	Support
H4a: Perceptual behavioral control → actual behaviour	0.036	0.080	ns	Not supported
H5: behavioral intention → actual behaviour	0.111	0.710	***	Support

***p < 0.001, **p < 0.01, *p < 0.05, ns p > 0.1.

Data source: Compiled by the author from AMOS software runs.

We further test hypothesis H4b by mediating effects: consumers' relevant perceived control behavior can influence actual purchase behavior through the mediating variable behavioral intention. Zhang et al.^37^ proposed that the conditions for testing the mediating effect are as followed: (1) regressing the independent variable on the dependent variable with a significant coefficient *a*, which is a prerequisite for the mediating effect; (2) regressing the independent variable on the mediating variable with a significant coefficient *b*, which means there is an effect of the independent variable on the mediating variable; (3) after controlling for the mediating variables and regressing the independent and mediating variables on the dependent variable simultaneously, the resulting regression coefficient *c* of the mediating variable on the dependent variable is significant, while the regression coefficient *d* of the independent variable on the dependent variable is insignificant, or the effect size is significantly reduced relative to *c*. The presence of mediating effect is determined when the above three conditions are met simultaneously. The coefficient *d* is used to determine whether the mediating effect is partially mediated (*d* is significant) or fully mediated (*d* is not significant). In this paper, all the observed variables involved in perceived behavioral control, behavioral intention, and actual behavior were averaged and centered, and then used SPSS 25.0 to analyze "dependent variable to independent variable", "mediator variable to independent variable", "dependent variable to independent variable and mediator variable". Finally, the existence and the type of mediating utility were judged based on the significance of the regression coefficients, and the results are shown in Table 10.

**Table 10:** Regression equations to test the mediating effect of behavioral intention as a function of perceived behavioral control and actual behavior.

IV	M	DV	IV → DV	IV → M	IV + M → DV		Types of mediating effects of *M*
					IV → DV	M → DV	
Perceived behavioral control	Behavioral intention	Actual behavior	0.357***	0.334***	0.050***	0.525***	Partial mediation

***p < 0.001, **p < 0.01, *p < 0.05, ns p > 0.1.

Data source: the authors compiled the results based on SPSS25.0 software runs.

The results indicated that perceived behavioral control indirectly influences actual behavior through the mediating variable of behavioral intention. The calculated contribution of the mediating effect to the total effect was 49.12%, and the mediating effect explained 24.4% of the variance variance of the dependent variable.

Xian et al.^38^ concluded that individual characteristics such as gender and age have significant effects on green consumption behavior. Due to this, this paper constructed a multicluster model based on gender, age, income, and education to analyze the psychology attributions of different types of consumers' new energy hybrid vehicle purchase behavior. We hope the results will suggest corresponding policy insights for the effective promotion of new energy vehicles.

As can be seen from Table 11, the RMSEA values for each characteristic met the criterion of < 0.05. Among the absolute fit, value-added fit and parsimonious fit statistics, all of the individual characteristics met the acceptable criteria of the model, except for the PGFI for education (0.492) and the NFI for age (0.868), which did not meet the criteria. This indicates that the theoretical model for the different individual characteristics in this paper fits well with the survey data, and the convergence validity of the multi-group model is quite good and can be used to test the research hypothesis. In this paper, the hypotheses of this paper are tested one by one through a comparative analysis of the multi-cluster models of individual consumer characteristics. The results are shown in Table 12.

**Table 11:** Summary table of the overall model fitness test for the multi group structural model.

Statistical test volume	Standard	Gender	Age	Revenue	Academic qualifications	Transportation consumption
Absolute suitability index						
** χ**^**2**^	p > 0.05	76.354	115.311	62.788	62.638	56.279
		p = 0.158	p = 0.067	p = 0.089	p = 0.052	p = 0.221
Cardinality ratio of freedom	< 2.00	1.175	1.227	1.281	1.362	1.149
RMR value	< 0.05	0.035	0.049	0.039	0.034	0.040
RESEA value	< 0.08	0.022	0.025	0.028	0.032	0.020
GFI value	> 0.90	0.956	0.934	0.961	0.962	0.966
AGFI value	> 0.90	0.939	0.905	0.928	0.926	0.937
Value added suitability index						
NFI value	> 0.90	0.910	***0.868***	0.926	0.926	0.933
IFI value	> 0.90	0.985	0.973	0.983	0.979	0.991
TLI value	> 0.90	0.984	0.968	0.974	0.966	0.986
CFI value	> 0.90	0.985	0.972	0.982	0.978	0.990
Minimalist suitability index						
PGFI value	> 0.50	0.690	0.654	0.523	***0.492***	0.526
PNFI value	> 0.50	0.821	0.755	0.631	0.591	0.635
PCFI value	> 0.50	0.890	0.846	0.668	0.625	0.674
CN value	> 200	442	399	426	406	475

Data source: The authors compiled the results based on AMOS software runs.

Significant values are in [italiced bold].

**Table 12:** Comparative analysis of multiple cohort models based on individual consumer characteristics.

Assumptions	Gender		Age			Academic qualifications		Revenue		Transportation consumption	
	Women	Male	Youth	Midlife	Elderly	Low education	High education	Low income	High income	Low consumption	High consumption
H1: Behavioral attitude → Behavioral intention	0.396***	0.469***	0.438***	0.443***	0.493***	0.445***	0.480***	0.442***	0.454***	0.526***	0.366***
H2: Subjective norm → Behavioral intention	0.363**	0.274***	0.301***	0.356**	0.261**	0.294***	0.317***	0.305***	0.313***	0.301***	0.326***
H3: perceived behavioral control → behavioral intention	0.325**	0.444***	0.342**	0.498**	0.319**	0.377***	0.406***	0.388***	0.398***	0.384***	0.385***
H4a: perceived behavior control → actual behavior	0.087 (ns)	0.092 (ns)	0.144 (ns)	0.191 (ns)	0.217 (ns)	0.062 (ns)	0.353 (ns)	0.109 (ns)	0.117 (ns)	0.109 (ns)	0.115 (ns)
H5: Behavioral intention → actual behavior	0.679***	0.730***	0.591***	0.539**	0.660***	0.728***	0.724***	0.680***	0.716***	0.672***	0.710***

Samples aged 29 and below are classified in the youth group, those aged 30–59 are classified in the middle-aged group, those aged 60 and above are classified in the elderly group; samples with education level of college and below are classified in the low education group, those with education level of bachelor and above are classified in the high education group; samples with monthly income of RMB 2999 and below are classified in the low income group, those with income of RMB 3000 and above were classified in the high income group; samples with an average monthly transport cost of RMB400 and below were classified in the low consumption group, and those with a transport cost of RMB401 and above were classified in the high consumption group.

***p < 0.001, **p < 0.01, *p < 0.05, ns p > 0.1.

Data source: The authors compiled the results based on AMOS software runs.

Significant values are in [bold].

In terms of significance level, the model tests for consumers with different individual characteristics satisfy hypotheses H1, H2, H3, and H5. The magnitude of the effect coefficients shows that compared to male, low-educated, low-income, low-transportation-consuming, and youth consumers (β = 0.274, 0.294, 0.301, 0.305, 0.301), females, highly educated, high transport consumption, high income, middle-aged consumers (β = 0.363, 0.317, 0.326, 0.313, 0.356) had a greater degree of influence of subjective norms on behavioral intentions. This may be because, when buying a new energy hybrid vehicle, these consumer groups are extremely externally focused and are more impacted by social groups than by elements like consumer behavioral control and vehicle perception. For instance, middle-aged, high-consumption consumers frequently must choose their own consumption habits based on the same circle of groups as their own due to their position or employment requirements. He et al.^39^ discovered that low-income groups were more receptive to and interested in incentives than other groups, and that persons with higher education levels tended to advocate the purchase of hybrid automobiles^40^. Also, men are more logical than women, who are more likely to be driven to buy eco-friendly products^41^. Female consumers, on the other hand, are more interested in premium or recently introduced products and are more fashion aware. In comparison to conventional vehicles, modern energy hybrid vehicles are newer in terms of configuration and performance. Customers will be more likely to select to buy new energy hybrid cars, indicating a stronger desire to buy, if doing so will increase their sense of superiority over other community members and make them feel better about themselves. For extroverted buyers, a sense of social acceptance or moral superiority dominates their impression of the value of a new energy hybrid vehicle.

The influence of behavioral attitudes (β = 0.469, 0.445, 0.526, 0.442, 0.438) on behavioral intentions was significantly higher for male, low education, low transportation consumption, low income, and youth consumers than the influence of subjective norms (β = 0.274, 0.294, 0.301, 0.305, 0.301) on behavioral intentions. It's possible that buying a new energy hybrid car is an expensive proposition for young consumers, the majority of whom are still in school. As a result, they will be more focused on the cost effectiveness of the vehicle when considering making the purchase. Consumers with low income, low education levels, and low levels of transportation spending will prioritize determining if the advantages of purchasing a new energy hybrid automobile outweigh those of using public transit. Men are more logical while shopping, thus they will consider the cost-effectiveness of significant outlays on cars. As a result, these buyers are incredibly introspective and have a tendency to follow their hearts while looking for a hybrid car. As opposed to outside expectations or interference, they prefer to follow their hearts and concentrate on whether a product's performance is consistent with their own. For instance, this consumer segment is concerned with the product's pricing. Consumers who are more reserved tend to value new energy hybrid vehicles more due to economic considerations.

Although the effects of perceived behavioral control on behavioral intention were all significant, its coefficient of influence on behavioral intention was lower than that of behavioral attitude and subjective norm. This may be due to the high recognition of new energy hybrid cars in life. On April 18, 2016, in order to better distinguish and identify new energy hybrid cars and implement differentiated traffic management, China launched a special number plate for new energy hybrid cars. New energy license plate in the color of green as the main color, reflecting the "green environmental protection" implication. And new energy hybrid car number plate to increase the special logo, the logo as a whole to green as the background color, meaning electric, new energy, green circle in the right side of the electric plug pattern, the left side of the colorful part and the English letter "E" (Electricity) similar. Therefore, most of the people can quickly identify the new energy hybrid cars. Consumers' perception of the role of new energy vehicles in environmental management is biased^42^. In addition, none of the perceived behavioral controls for different individual characteristics had a significant effect on actual behavior, but behavioral intention still had a significant positive effect on actual behavior.

## Conclusions and recommendations

### Research findings

Firstly, based on the theory of planned behavior and the mediating effect, we studied the psychological attribution of consumers' new energy hybrid electric vehicle purchase behavior, we drawed the following conclusions: there are significant positive effects of perceived behavioral control, behavioral attitude, and subjective norm on behavioral intention, and behavioral intention also has significant effects on actual behavior. But there is no significant effect of consumers' perceived behavioral control on actual purchase behavior, it indirectly influences actual behavior through the mediating variable of behavioral intention.

In order to understand the psychological attributions of different types of consumers when purchasing new energy hybrid vehicles, this paper further constructed a multi-cluster model to analyze the findings of different individual characteristics of consumers. The findings are as follows: for extroverted consumers (female, middle-aged, high transportation consumer, high education, high income), they are more likely to be influenced by other members of the society around them when deciding to purchase a new energy hybrid vehicle, and they are more concerned with the feeling of superiority or status enhancement brought by the purchase than the consumers' own need or knowledge of the product. For introverted consumers (male, low education, low transportation consumption, low income, and youth), they care more about the cost effectiveness of the car when shopping, and the influence of behavioral attitude on behavioral intentions is significantly higher than the influence of subjective norm on behavioral intentions. However, for both introverted and extroverted consumers, the coefficients of perceived behavioral control on behavioral intention were slightly lower than those of behavioral attitude as well as subjective norm, while behavioral intention still had a significant positive effect on actual behavior.

### Policy recommendations

Based on the above findings, this paper offers the following policy insights.

1. Develop differentiated communication programs
  The variables determining how consumers of different genders, ages, educational levels, and income levels use green products vary^43^. The timing of new energy vehicle purchases by consumers is influenced by a number of variables, including education, annual income, government legislation, and peer judgments^44^. Hence, targeted marketing initiatives for customers with various traits might encourage customer buy behavior. This study classified female, high education, high transportation consumption, high income, and middle-aged customers as extroverted consumers, and these consumer groups had a strong outward orientation in their spending patterns, according to the findings of the cohort structure model analysis. Men, people with low levels of education, poor transportation, people with little money, and those in their twenties are categorized as having an inward-looking consumer behavior. It is evident from the preceding analysis that there are differences between the characteristics that influence introverted and extroverted consumers' propensity to buy new energy hybrid automobiles. Being environmentally conscious will increase a person's likelihood of buying a hybrid vehicle because previous government publicity on hybrid vehicles has centered on their environmental friendliness and extroverted consumers are more interested in the sense of superiority that a hybrid vehicle can bring. It's vital to remember that introverted consumers are currently more interested in the advantages of the new hybrid vehicle itself than they are in the environmental benefits of green products. More can be done to highlight the advantages of acquiring new energy hybrids, such as purchase incentives, extended warranties, etc., when promoting these vehicles. Tax credits for new-energy vehicles can lower consumers' tax rates on vehicle purchases^45^. In parallel, specific industry norms are being established to further safeguard consumers' rights and interests and allay their concerns around the purchase of new energy hybrid vehicles.
2. Improving the performance of new energy hybrid vehicles
  Subjective norm have a significant positive effect on behavioral intentions. New energy hybrid vehicles as a new industry, many consumers may not know much about new energy hybrid vehicles, and extroverted consumers are more inclined to the opinions and views of people around them. Therefore, in addition to increasing the publicity and promotion of new energy hybrid vehicles, it is more important to improve the quality of new energy hybrid vehicles, optimize performance, and establish a good reputation among consumers who have already purchased new energy hybrid vehicles in order to win more consumer recognition.
3. Reduce the development cost of new energy hybrid vehicles
  Although new energy hybrid vehicles have the advantage of energy saving and environmental protection, their high annual maintenance cost becomes an important factor that hinders the market sales volume^46^. At present, many consumers are reluctant to purchase new energy hybrid vehicles because of the high price and the subsequent maintenance costs are unaffordable. For example, the previously mentioned introverted consumers, who buy new energy hybrid vehicles, care more about the performance of the product and whether the price/performance ratio meets their expectations, and the pricing factors that are classified as expensive hybrid vehicles can lead to the fading of positive attitudes toward the purchase of the vehicle^47^. If the price of hybrid vehicles can be reduced to an affordable price, it may attract the interest of people with positive attitude towards hybrid vehicles. Therefore, this paper suggests that companies should actively develop new technologies to reduce production costs, so that the price of new energy hybrid cars down; establish an industry standard related to the development of plug-in hybrid electric vehicles, so that their related components are common and accessories form a large-scale production, thereby reducing vehicle prices; at the same time, the government should also increase subsidies to further reduce the cost of new energy hybrid vehicles purchased by consumers.

## Limitations

This study uses a classic theory from social psychology to analyse psychological attributions and intervention strategies for new energy hybrid vehicle purchase behaviour through questionnaire and software analysis. However, this paper is a cross-sectional related study, which is a key limitation of this paper. Cross-sectional studies, also known as cross-sectional surveys, because the descriptive information obtained is collected at a point in time or over a short time interval, it objectively reflects the relationship between variables at this point in time and cannot be used to analyze behavior over time or to establish long-term trends. Therefore, the findings of this study can only be used as exploratory findings, which are only applicable to describe and explore the relationship between variables of the survey respondents during the survey period, and cannot be used to prove causality. The policy recommendations presented in this paper can only be used as a reference for policy-making authorities in the surveyed areas. Specifically, actual purchase behaviour may in turn reinforce consumers' willingness to engage in such behaviour again in the future, while behavioural intentions may provide motivation for actual purchase behaviour in the form of favourable behavioural attitudes, subjective norms and perceived behavioural control. For this reason, in future research, more attention should be paid to the need for data collection to be conducted earlier, to lengthen the data collection period and to obtain data from different time periods to ensure that the data are suitably unbiased.

## Data Availability

The data and estimation commands that support the findings of this paper are available on request from the first and corresponding authors.
